# The membrane may be a key factor influencing browning: a mini review on browning mechanisms of fresh-cut fruit and vegetables from a multi-omics perspective

**DOI:** 10.3389/fnut.2025.1534594

**Published:** 2025-02-25

**Authors:** Xiao Yuan, Zhaoxia Zhan, Wei Lin, Can Zhang, Bin Wang

**Affiliations:** ^1^Guangdong Provincial Key Laboratory of Utilization and Conservation of Food and Medicinal Resources in Northern Region/College of Biology and Agriculture, Shaoguan University, Shaoguan, China; ^2^Guangdong Provincial Engineering and Technology Research Center of Special Fruit and Vegetables in Northern Region, Shaoguan University, Shaoguan, China

**Keywords:** ready-to-eat food, fresh-cut fruit and vegetables, enzymatic browning, cell membrane, multi-omics research

## Abstract

Fresh-cut fruit and vegetables are susceptible to browning during storage and subsequent consumption. The cell membrane acts as a vital structural barrier, compartmentalizing various substances within living organisms. The fresh-cutting process induces mechanical injuries, disrupting these membranes and resulting in the leakage of cellular contents. This facilitates direct contact between substances and enzymes that mediate browning reactions. This mini review explores the potential roles of cell membranes in the browning of fresh-cut fruit and vegetables from a multi-omics perspective, aiming to provide novel insights into the underlying mechanisms of browning in fresh-cut fruit and vegetables. Considering potential roles of cell membranes in blocking the browning of fresh-cut fruit and vegetables, future studies should focus on elucidating the precise mechanisms by which membranes regulate browning reactions, aiming to provide directions for the development of more effective intervention strategies.

## 1 Introduction

Fresh-cut fruit and vegetables are a convenient form of ready-to-eat fresh food (1). Their appeal lies in their convenience, freshness as well as high edible rate, leading to a surge in popularity among consumers (2). However, these products are highly susceptible to browning after peeling or cutting, a process that not only diminishes their visual appeal but also reduces their nutritional value and shelf life (3).

The browning in fresh-cut products can be classified into enzymatic and nonenzymatic types (4, 5). Enzymatic browning is primarily driven by phenolase enzymes, particularly polyphenol oxidase (PPO) and peroxidase (POD) (6). In contrast, nonenzymatic browning occurs independently of phenolase and may include processes such as ascorbic acid oxidation, the Maillard reaction, and caramelization (7). For example, browning in most fruit or vegetable species, such as pear, apple, and potato, is enzymatic (8–10). However, in other species such as Chinese water chestnuts, the absence of phenolase substrates leads to browning after peeling (11, 12). These studies indicate that the mechanisms underlying browning in fresh-cut products are highly complex, involving both enzymatic and nonenzymatic processes.

Therefore, understanding the browning mechanisms of fresh-cut fruit and vegetables is essential for developing effective preservation strategies. While numerous studies have concentrated on understanding the browning mechanisms at both physiological and molecular levels, the roles of cell membrane influencing this process have garnered less attention. Multi-omics is an integrative approach that offers a comprehensive understanding of complex biological processes by simultaneously analyzing various types of omics data (13), such as genomics, transcriptomics, proteomics, and metabolomics. In this review, we explored the potential roles of cell membranes in the browning process of fresh-cut products from a multi-omics perspective, aiming to provide valuable insights for developing strategies to mitigate browning.

## 2 The roles of membranes in blocking the browning of fresh-cut produce

The cell membrane of eukaryote is a complex structure that functions as a selective barrier, regulating the influx and efflux of substrates (14). It is primarily composed of a diverse array of lipids and proteins (15). Under normal conditions, substrates and enzymes involved in browning are compartmentalized within distinct subcellular organelles by biological membranes (16). Fresh-cut operations, such as peeling or cutting, compromise cell integrity and disrupt compartmentalization (17). As a result, components that were previously separated within distinct subcellular organelles come into direct contact, triggering the browning process.

**Figure 1:**
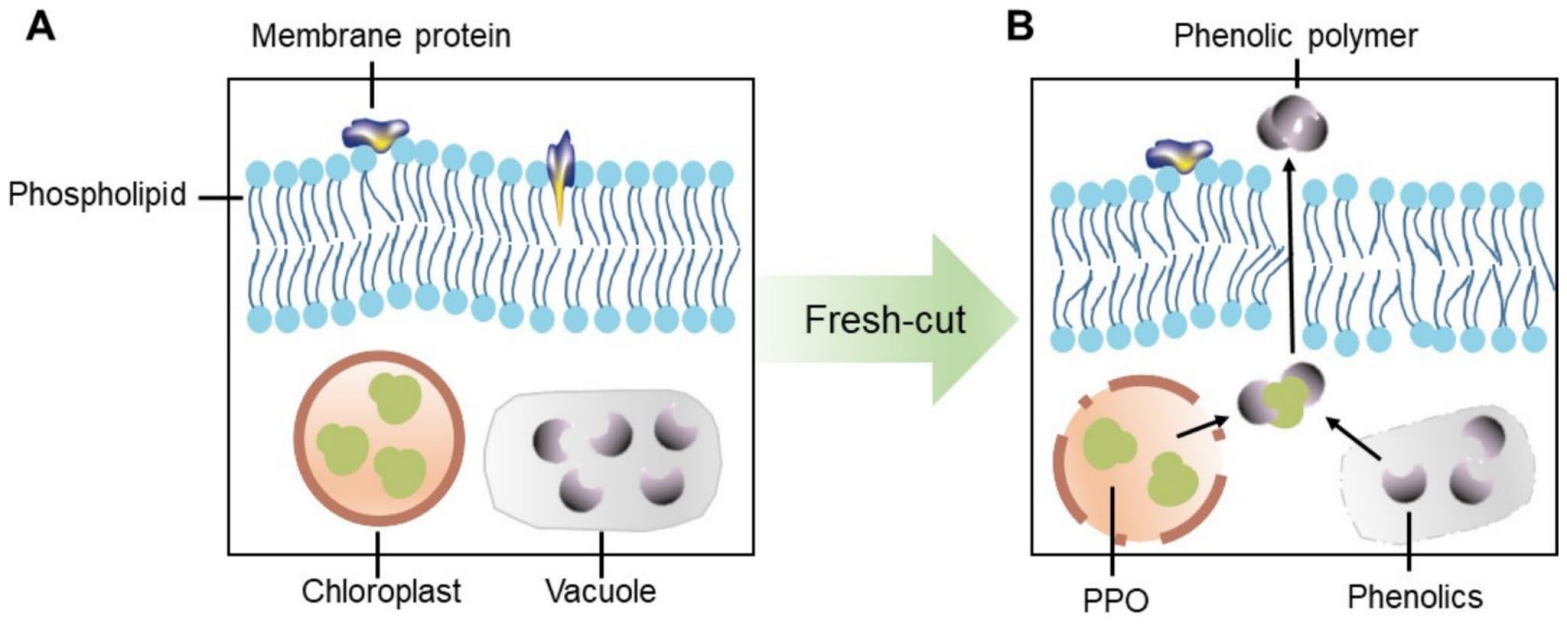
A proposed model illustrating how the cell membrane functions to block the interaction between phenolics and phenolases such as PPO. **(A)** Phenolics and phenolases are separated under normal condition. **(B)** Phenolics and phenolases are directly contacted after fresh-cutting.

PPO and POD are two key enzymes that contribute to the oxidation of phenolic substrates in enzymatic browning. This process occurs through the enzymatic oxidation of monophenols or o-diphenols, leading to the production of o-quinones, which then undergo subsequent condensation or polymerization reactions (18). PPO, primarily located in chloroplasts, catalyzes the oxidation of various phenolic compounds to o-quinones (19). In contrast, POD, which is potentially found in cell walls, vacuoles, and apoplasts (the space outside the plasma membranes), functions in the oxidoreduction of hydrogen peroxide and various reductants (16). Most phenolic compounds, however, are stored in vacuoles (18). The loss of cellular compartmentalization allows PPO and/or POD to come into direct contact with phenolics, leading to the oxidation of phenolics and the formation of brown polymers (20). The above discussions indicate that the degradation of membranes occurs prior to the browning reactions, highlighting the role of membranes in regulating this process. Building on this hypothesis, we propose a model to illustrate how the cell membrane functions to block the interaction between phenolics and phenolase such as PPO (Figure 1).

Biological membranes are characterized by a phospholipid bilayer (21). Numerous studies have demonstrated that fresh-cut operations stimulate the activity of lipid metabolism enzymes, including phospholipase (PL) and lipoxygenase (LOX), leading to the degradation of membrane lipids. PLs catalyze the initial steps of lipid hydrolysis, while LOXs facilitate the oxygenation of polyunsaturated fatty acids, leading to the peroxidation of membrane lipids (22). The relationship between browning and the activity of PLD and LOX has been documented in fresh-cut potato (23), taro (24), and eggplant (25).

Furthermore, fresh-cutting triggers a burst of reactive oxygen species (ROS), which contributes to the oxidation of biomacromolecules, particularly membrane lipids. This oxidative stress leads to functional defects in membranes, including altered permeability and stability (26). Moreover, elevated ROS levels may also act as signaling molecules, mediating browning reactions (27). For instance, melatonin (MT) has been shown to effectively reduce browning in various fresh-cut food species (3). Concurrently, the increased ROS levels induce the production of endogenous MT, thereby influencing the redox homeostasis of fruit and vegetables during the postharvest period (28).

## 3 Multi-omics approaches used to reveal the involvement of membrane in browning of fresh-cut produces

### 3.1 Genomics analysis

Genomics analysis examines genetic variability within species and identifies genes or pathways closely associated with specific traits (29). However, researches aimed at identifying key genes or modules that regulate the browning of fruit and vegetables remains limited to date. Most studies have concentrated on genome-wide analyses or genome-wide association studies (GWAS). For instance, in banana (*Musa acuminata*), 10 *PPO* genes were successfully identified based on a high-quality genome sequence, with *MaPPO1* and *MaPPO6* identified as the primary contributors to fruit browning (30).

Thioesterase regulates the flow of various substrates by releasing CoA from intermediates and products of β-oxidation (31). In water yam (*Dioscorea alata*), two SNP markers located on chromosome 5 were significantly associated with oxidative browning of the tuber through GWAS, and suggested that genes with thioesterase domain may play an important role in tuber browning (32).

The enhanced LOX protein activity or gene expression leads to the peroxidation of membrane lipids and subsequent browning of fresh-cut produces (33). Treatments with methyl jasmonate (MeJA) induce effects similar to those of wounding stress (34), and the expression of two *LOX* genes, *MaLOX2-4* and *MaLOX9* identified through genome-wide analysis, was significantly induced following MeJA treatment in banana (35). Within the same species, some varieties are susceptible to browning while others are not. Therefore, researchers could compare the genetic variability among these varieties to identify key membrane-related contributors to browning through comparative genomics analysis.

### 3.2 Transcriptomics analysis

Transcriptomics analysis measures gene expression patterns to elucidate molecular changes at transcript level (36). Comparative transcriptomics is the most widely used omics approach for revealing the mechanisms underlying the browning of fresh-cut produces. This analysis can identify differentially expressed genes (DEGs) in response to fresh-cutting or during the browning process.

A previous study investigated the browning mechanism of fresh-cut eggplant by comparing transcriptomic differences between browning-resistant (‘F’) and browning-sensitive (‘36’) cultivars (24). The results showed that numerous DEGs and differentially accumulated metabolites (DAMs) were associated with lipid metabolism, as revealed by the integrated analysis of transcriptome and metabolome data, suggesting the increased expression of membrane lipid-degrading enzymes might accelerate the browning of fresh-cut eggplant. Preharvest selenium (Se) treatment significantly reduced the browning potential of fresh-cut apples. Transcriptome analysis revealed that genes involved in membrane lipid oxidation, such as *LOX* and *PLD*, were notably downregulated following Se treatment (37).

In fresh-cut taro, genes involved in the linoleic acid metabolic pathway were induced after peeling, with expression levels rising in tandem with aggravated browning (23). The application of cinnamic acid (CA) was found to effectively inhibit browning in cold-stored taro slices, and the downregulated DEGs resulting from CA treatment were significantly enriched in the membrane lipid metabolism related pathways, as revealed by comparative transcriptomics and weighted gene co-expression network analysis (38). These studies demonstrate that membrane lipid metabolism may play a critical role in the browning process of fresh-cut produces.

### 3.3 Proteomics‌ analysis

Proteomics analysis utilizes high-throughput technologies to comprehensively evaluate the qualitative and quantitative aspects of a sample’s entire protein repertoire (39). Compared to transcriptomic or genomic data, proteomic information provides more direct insights into the functional molecules involved in biological processes, since protein levels and activities cannot be fully predicted from RNA or DNA data alone (40).

Recent proteomic studies have investigated protein modifications during the browning process of fresh-cut produces. In ‘Fuji’ apple, KEGG enrichment analysis of differentially expressed proteins (DEPs) identified significant alterations in multiple metabolic pathways, including carbon metabolism, amino acid biosynthesis, and secondary metabolite biosynthesis. Moreover, authors had observed a significant increase in the abundance of O-methyltransferase 1 protein following browning in T-type apples (41). Using iTRAQ proteomics, researchers identified over 1900 DEPs when comparing yellow fresh-cut yam to white fresh-cut yam. The upregulated DEPs in yellow yam showed significant enrichment in several biosynthesis pathways, such as carotenoid biosynthesis and phenylpropanoid biosynthesis (42).

From a membrane lipid metabolism perspective, studies in pear fruit revealed that browning-related DEPs primarily participated in the linoleic acid and fatty acid biosynthesis pathways (43). In fresh-cut lettuce browning, researchers observed increased LOX activity during the browning process. Metabolomic and proteomic analyses further demonstrated that stem browning highly correlated with decreased unsaturated fatty acid content, while fresh-cut-induced ROS contributed to fatty acid oxidation (44). These studies further highlight the roles of cell membrane in the browning of fresh-cut produces.

### 3.4 Metabolomics analysis

Metabolites are small molecules that represent the end products of cellular processes and are crucial for understanding the biochemical state of living organisms (45). Metabolomics analysis identifies these metabolites using GC–MS or LC–MS technologies, and could employ comparative strategies to capture changes in metabolite levels among samples.

Metabolomics analysis has been widely applied to elucidate potential mechanisms in fresh-cut products. Comparative metabolomics studies have revealed that, during the browning process of fresh-cut apples, the accumulated metabolites are primarily phenols and lipids (37). Similarly, during the browning of fresh-cut taro, the abundance of 11 metabolites consistently increased, with 10 of them being linolenic acid and the derivatives, as well as hydroperoxides (24). These researches clearly indicate the involvement of lipid metabolism, particularly membrane lipid metabolism, in the browning process.

## 4 Conclusion

In summary, the membrane is a vital structural component of plant cells that significantly influences the browning of fresh-cut produces. By employing a multi-omics approach, researchers could gain deeper insights into the complex interactions among membrane disorder, signaling transduction, and biochemical reactions that lead to browning. These understandings would help the development of targeted interventions designed to effectively mitigate browning, ultimately enhancing the quality and shelf life of fresh-cut products.
